# Lack of detectable sex differences in the mitochondrial function of *Caenorhabditis elegans*

**DOI:** 10.1186/s12862-024-02238-x

**Published:** 2024-04-26

**Authors:** Dillon E. King, A. Clare Sparling, Abigail S. Joyce, Ian T. Ryde, Beverly DeSouza, P. Lee Ferguson, Susan K. Murphy, Joel N. Meyer

**Affiliations:** 1 Nicholas School of Environment, Duke University, 308 Research Drive, A304, Durham, NC 27708 USA; 2 Department of Obstetrics and Gynecology, Duke University Medical Center, Durham, NC USA; 3 Pratt School of Engineering, Duke University, Durham, NC USA; 4 Department of Pharmacology and Cancer Biology, Duke University, Durham, NC USA

**Keywords:** *C. elegans*, Sex differences, Mitochondria, Mitochondrial toxicity

## Abstract

**Background:**

Sex differences in mitochondrial function have been reported in multiple tissue and cell types. Additionally, sex-variable responses to stressors including environmental pollutants and drugs that cause mitochondrial toxicity have been observed. The mechanisms that establish these differences are thought to include hormonal modulation, epigenetic regulation, double dosing of X-linked genes, and the maternal inheritance of mtDNA. Understanding the drivers of sex differences in mitochondrial function and being able to model them in vitro is important for identifying toxic compounds with sex-variable effects. Additionally, understanding how sex differences in mitochondrial function compare across species may permit insight into the drivers of these differences, which is important for basic biology research. This study explored whether *Caenorhabditis elegans*, a model organism commonly used to study stress biology and toxicology, exhibits sex differences in mitochondrial function and toxicant susceptibility. To assess sex differences in mitochondrial function, we utilized four male enriched populations (N2 wild-type male enriched, *fog-2(q71)*, *him-5(e1490)*, and *him-8(e1498)*). We performed whole worm respirometry and determined whole worm ATP levels and mtDNA copy number. To probe whether sex differences manifest only after stress and inform the growing use of *C. elegans* as a mitochondrial health and toxicologic model, we also assessed susceptibility to a classic mitochondrial toxicant, rotenone.

**Results:**

We detected few to no large differences in mitochondrial function between *C. elegans* sexes. Though we saw no sex differences in vulnerability to rotenone, we did observe sex differences in the uptake of this lipophilic compound, which may be of interest to those utilizing *C. elegans* as a model organism for toxicologic studies. Additionally, we observed altered non-mitochondrial respiration in two *him* strains, which may be of interest to other researchers utilizing these strains.

**Conclusions:**

Basal mitochondrial parameters in male and hermaphrodite *C. elegans* are similar, at least at the whole-organism level, as is toxicity associated with a mitochondrial Complex I inhibitor, rotenone. Our data highlights the limitation of using *C. elegans* as a model to study sex-variable mitochondrial function and toxicological responses.

**Supplementary Information:**

The online version contains supplementary material available at 10.1186/s12862-024-02238-x.

## Background

Mitochondria are complex organelles best known for their role in generating ATP through oxidative phosphorylation. Mitochondria are also central to signaling pathways involved in apoptosis, ion homeostasis, immune responses, and metabolic regulation [1, 2]. Disruptions in mitochondrial function can cause severe adverse effects and have been associated with many diseases [3]. Differences in mitochondrial function and metabolism have been observed between sexes in multiple species across a variety of tissue types, as have sex differences in sensitivity to certain mitochondrial toxicants [4–13]. Though this area of research is still in early stages [14], the general pattern emerging is that females may have slightly elevated mitochondrial function and may be more resilient to mitochondrial insults [15]. Though these sex differences appear to be subtle, they have the potential to alter health and disease susceptibility [15–17]. The mechanisms that establish these sex differences are not well understood, though multiple studies have pointed to hormones as key drivers of sex differences in mitochondrial function [16]. Other research indicates that epigenetic mechanisms [18] and double dosing of genes on the X-chromosome may be involved [19–21]. It has also been suggested that the maternal inheritance of mitochondrial DNA (mtDNA), which might enhance selective processes that drive the evolution of the mitochondrial genome in a female nuclear background, could result in elevated mitochondrial function in females [15, 22].

*Caenorhabditis elegans* is an established model organism whose mitochondrial biology is well-conserved with humans [23]. The mitochondrial genome of *C. elegans* is highly conserved, containing 12 genes that encode oxidative phosphorylation subunits, two ribosomal RNAs, and 22 tRNAs, versus 13, two, and 22 in humans, respectively. *C. elegans* is a genetically tractable model for which a wide range of functional mitochondrial and organismal fitness assays exist. Though *C. elegans* are predominately hermaphroditic, males exist at low frequencies (~ 0.2%). The proportion of males, however, increases during times of stress and following sexual reproduction [24]. Like humans, mtDNA in *C. elegans* is passed on exclusively through the oocyte, as mitochondria in the sperm are actively degraded upon fertilization [25, 26]. Thus, *C. elegans* may be used to study whether maternal mtDNA inheritance contributes to sex differences in mitochondrial function. Though sex differences in mitochondrial function have been reported in a variety of species, we are not aware of any reports characterizing differences by sex in the mitochondrial function of *C. elegans*. However, there is growing evidence for sex differences in both metabolism and disease responses in *C. elegans* [27–31].

A practical reason that it is important to understand potential sex differences in the mitochondrial function of *C. elegans* is the increasing use of this species for studies of mitochondrial diseases and toxicological testing. Mitochondrial health is associated with a variety of diseases [32] and many diseases that are hallmarked by mitochondrial dysfunction exhibit sex differences in prevalence [15]. Additionally, numerous pharmaceuticals and environmental compounds target mitochondria and it has been estimated that up to 15% of pollutants are mitochondrial toxicants [33–36]. Given their ease of culturing, fast reproduction time, and genetic tools, *C. elegans* are a powerful in vivo system to study mitochondrial biology and toxicology [37–43].

While mammalian studies have served as the “gold standard” in toxicology, mammalian experiments are expensive and time consuming. This is of significant concern given that there is little to no toxicological data for thousands of chemicals on the global market [44]. Furthermore, both the US Environmental Protection Agency and the European Union have announced plans to phase out mammalian toxicity studies and thus other models are needed. While *C. elegans* has the potential to serve as an important model system in toxicity testing, it is unclear how well *C. elegans* based screening can identify toxic compounds whose properties exert sex-specific effects.

In the present study, we assessed potential sex differences in mitochondrial function and susceptibility to a mitochondrial toxicant in *C. elegans*. We utilized *C. elegans* strains with varying proportions of males in their populations to study parameters of mitochondrial function such as whole worm respirometry, whole worm ATP levels, mtDNA copy number, as well as growth outcomes and uptake of a well-established Complex I inhibitor, rotenone. Our prediction was that the presence of any sex-specific differences in mitochondrial function would be evident when comparing populations with a higher proportion of males to typical *C. elegans* populations comprising predominantly hermaphrodites. However, this study indicates little to no detectable sex differences in mitochondrial fitness or susceptibility to mitochondrial stress, which fails to provide evidence for the role of maternal mtDNA inheritance patterns in establishing sex differences in mitochondrial function and highlights important limitations of using *C. elegans* as a toxicological screening tool.

## Results

**Fig. 1:**
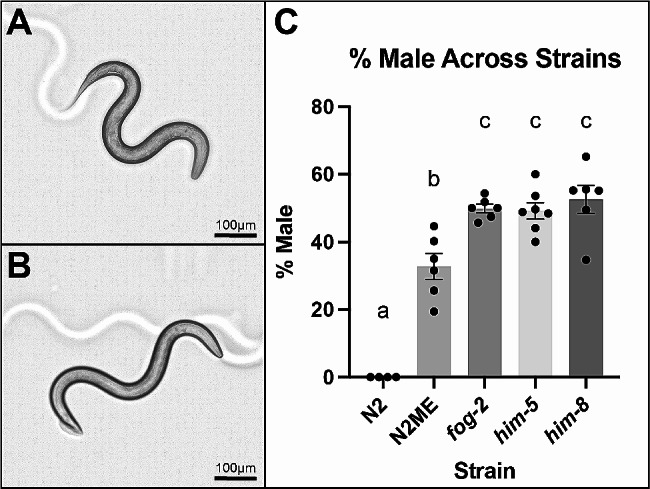
Characteristics of *C. elegans* sexes and strains used in this study. **(A)** Representative image of hermaphrodite *C. elegans* at the L4 stage. **(B)** Representative image of male *C. elegans* at the L4 stage. Images were taken at 10X using a Keyence BZ-X710. The scale bar represents 100 μm. **(C)** Proportion of males in each strain used in this study. Statistical significance of *p* < 0.05, determined by a one-way ANOVA with a Tukey post hoc test for multiple comparisons, is represented as (a) denoting the comparison of N2 to other strains, (b) denoting the comparison of N2 male enriched (N2ME) to other strains, and (c) denoting the comparison between *fog-2*, *him-5*, and *him-8* (N2ME vs. *him-8*: *p* = 0.0006, N2ME vs. *him-5*: *p* = 0.0032, N2ME vs. *fog-2*: *p* = 0.0032, N2ME vs. N2: *p* < 0.0001, *him-8* vs. *him-5*: *p* = 0.9163, *him-8* vs. *fog-2*: *p* = 0.9656, *him-8* vs. N2: *p* < 0.0001, *him-5* vs. *fog-2*: *p* = 0.9998, *him-5* vs. N2: *p* < 0.0001, *fog-2* vs. N2: *p* < 0.0001). Six biological replicates were assessed, each with > 75 worms assessed per replicate

In the wild type laboratory strain of *C. elegans*, N2 Bristol, males arise at low frequencies (0.2%) [45]. Males can be easily differentiated from hermaphrodites at the L4 stage by their thinner body, fanned tail, and lack of a vulva (Fig. 1A and B). Currently, there are no established methods to generate large populations of pure male *C. elegans.* To circumvent this issue, we utilized male enriched strains to study sex differences in mitochondrial function. Researchers can take advantage of heat shocking the worms to generate a population of roughly 2–5% males, as this increases meiotic nondisjunction [46]. Once a small number of males have been obtained, male enriched populations can be maintained by setting up mating plates [47], as 50% of *C. elegans* offspring generated from mating are males. To generate a large enough population of N2 male enriched (N2ME) worms, we used synchronized offspring from the F2 generation of mating plates, which resulted in a population of ~ 32% males (Fig. 1C). Additionally, we used genetically modified *fog-2(q71)*, *him-5(e1490)*, and *him-8(e1498)* strains, all of which maintain populations with ~ 50% males (Fig. 1C).

**Fig. 2:**
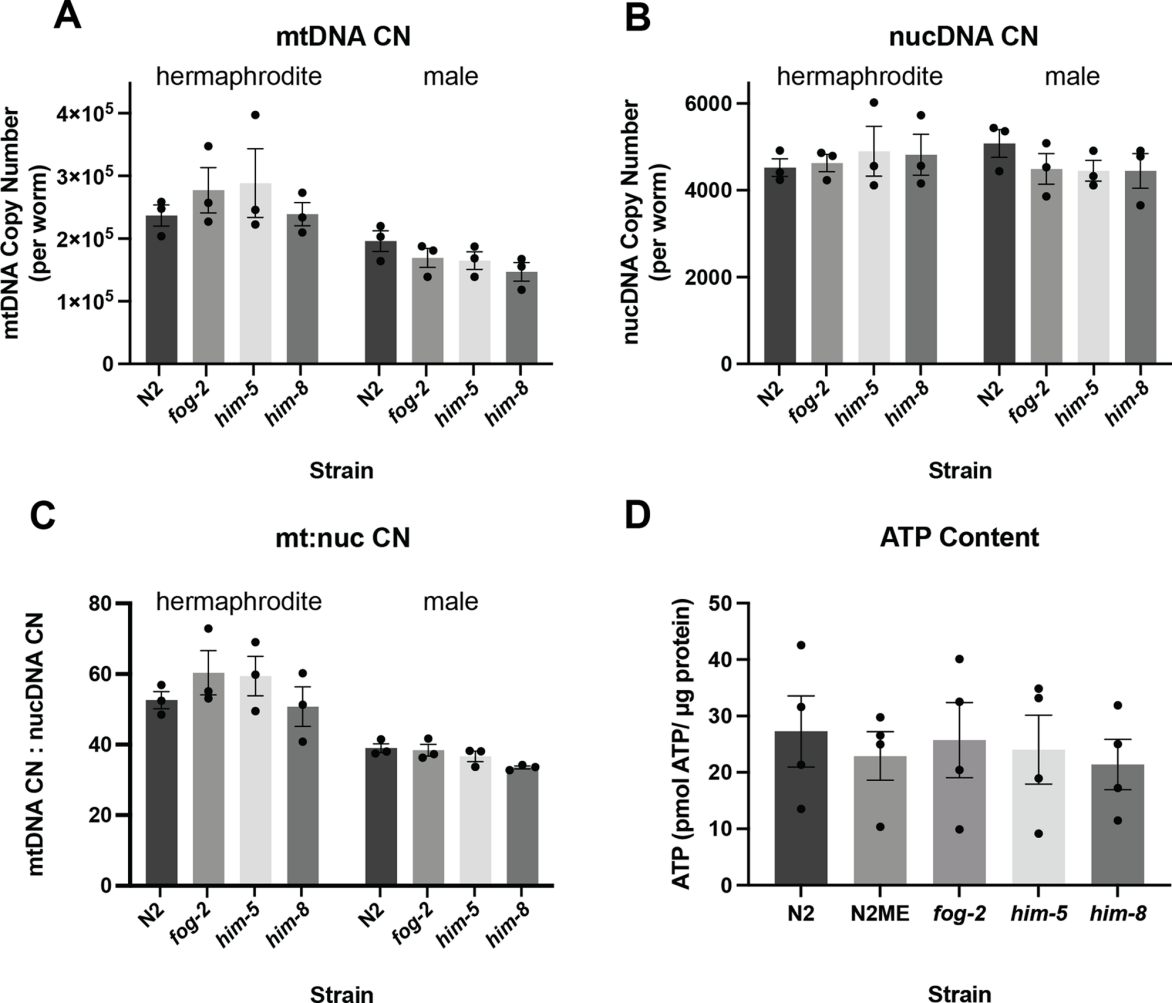
Mitochondrial parameters of male enriched strains. For all copy number values, copy number is expressed per worm. **(A)** mtDNA copy number (CN) of each strain, separated by sex. For copy number analysis, three biological replicates were performed. Data was assessed by two-way ANOVA for both sex and strain. Sex: *p* = 0.0002***, strain: *p* = 0.6079, interaction: *p* = 0.4746. **(B)** nuclear genome (nucDNA) copy number of each strain, separated by sex. Data was assessed by two-way ANOVA for both sex and strain. Sex: *p* = 0.7707, strain: *p* = 0.9277, interaction: *p* = 0.5256. **(C)** mtDNA: nucDNA copy number ratio of each strain, separated by sex. Data was assessed by two-way ANOVA for both sex and strain. Sex: *p* < 0.0001***, strain: *p* = 0.2810, interaction: *p* = 0.6026. **(D)** Mean ATP levels of each strain, in pmol of ATP per microgram of protein in each strain. For ATP level analysis, four biological replicates were performed. Data was assessed by a one-way ANOVA, *p* = 0.9625

We observed sex differences in mtDNA copy number (*p* = 0.002) and mtDNA copy number relative to nuclear DNA copy number (*p* < 0.0001), but not in nuclear DNA (nucDNA) copy number (*p* = 0.7707) (Fig. 2A-C). However, the difference in mtDNA copy number is expected and not likely a result of sex per se, but rather gonadal development; during the L4 stage, *C. elegans* begin to develop their germline and oocytes contain many copies of mtDNA [48]. Though sex differences were present within each strain, there were no differences in mtDNA copy number among the strains. Interestingly, no differences were observed in whole worm ATP levels, despite known differences in size and cell number between L4 males and hermaphrodites and our observed differences in mtDNA copy number (Fig. 2).

**Fig. 3:**
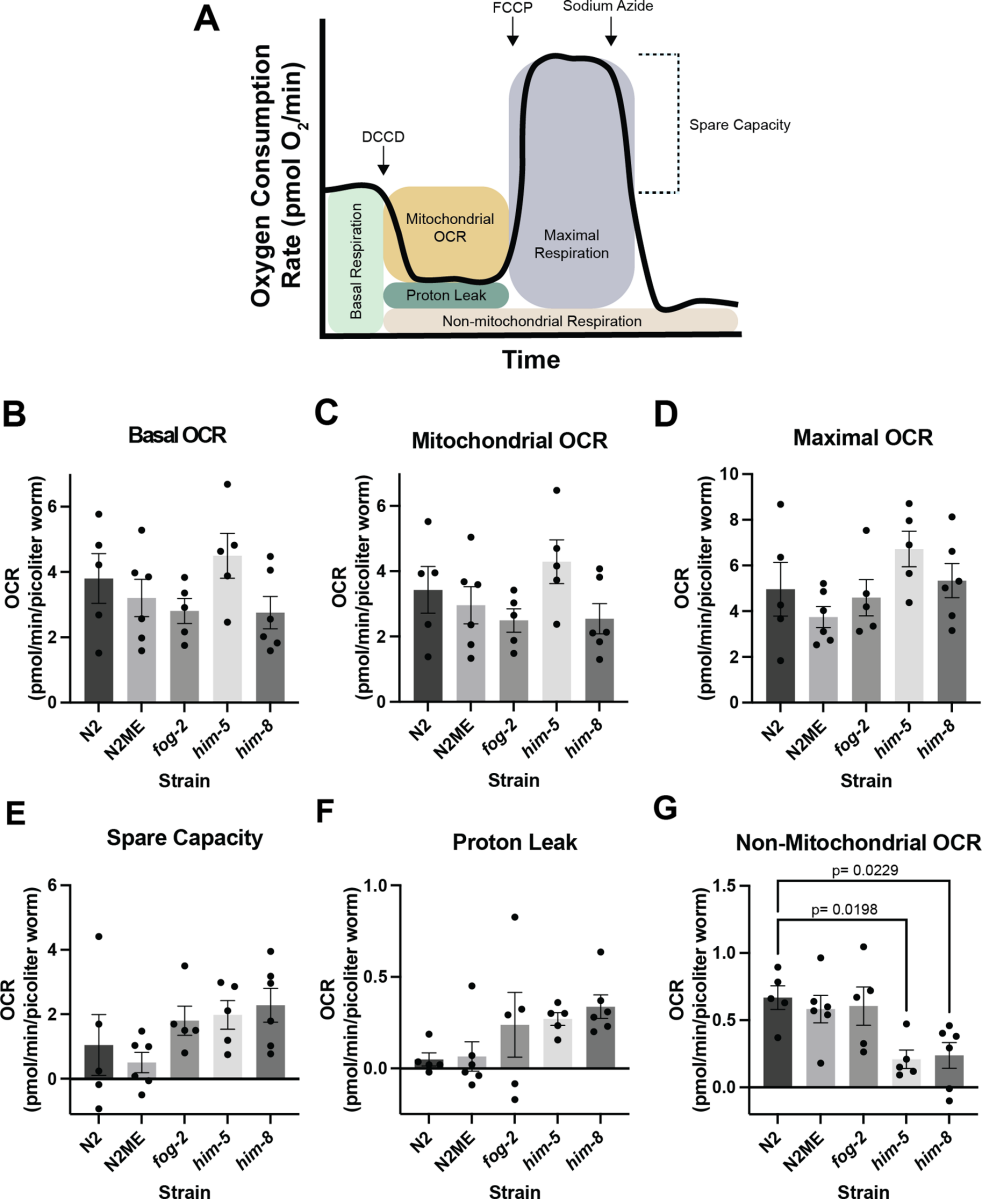
Seahorse respirometry analysis across male enriched strains. **(A)** Representative image of the mitochondrial function parameters assessed using the inhibitors dicyclohexylcarbodiimide (DCCD), carbonyl cyanide 4-(trifluoromethoxy) phenylhydrazone (FCCP), and sodium azide. The Seahorse XFe24 Analyzer determines the oxygen consumption rate (OCR) in pmol O_2_ per minute prior to and following injections of the compounds. **(B)** Mean basal OCR across strains. **(C)** Mean mitochondrial OCR across strains. **(D)** Mean maximal OCR across strains. **(E)** Mean spare capacity across strains. **(F)** Mean proton leak across strains. **(G)** Mean non-mitochondrial OCR across strains (N2 to *him-5p* = 0.0419, N2 to *him-8p* = 0.0481). For figures **B-G**, the x-axis displays the strain and the y-axis displays the OCR associated with each parameter in pmol/min, normalized to the volume of worm in each well in picoliters. Statistical significance of all pairwise comparisons were assessed using a one-way ANOVA with a Tukey post hoc test for multiple comparisons when normally distributed and a Kruskal-Wallis test with a Dunn’s post-hoc test for multiple comparisons when not normally distributed, as was the case for proton leak. Data represents five biological replicates per strain

We next assessed whole worm respirometry across strains under basal conditions and in response to chemical exposures of FCCP (a mitochondrial uncoupler), DCCD (an inhibitor of ATP synthase), and sodium azide (an inhibitor of Complex IV of the electron transport chain) (Fig. 3A). Basal oxygen consumption rate (OCR) is defined as the amount of oxygen being consumed by the whole worm at a resting state, while mitochondrial OCR represents the oxygen consumption utilized by the electron transport chain alone. Maximal respiration represents the organism’s maximal ability to consume oxygen [49]. Compared to N2 WT worms, which are < 0.02% male, male enriched strains showed no differences in basal, mitochondrial, or maximal OCR on the Seahorse Analyzer (Fig. 3B, C and D). Spare capacity provides a measure of the level at which an organism is functioning in relation to its maximal respiration, so an organism with low spare capacity is already respiring near its maximum capacity, and an organism with high spare capacity will be able to increase oxygen consumption in times of need [49]. Proton leak is the mitochondrial oxygen consumption associated with any processes other than oxidative phosphorylation. Elevated levels can be a sign of damage, or a mechanism to regulate temperature [50]. Both spare capacity and proton leak appear to increase with the proportion of males in the population, but do not reach statistical significance (Fig. 3E and F). Non-mitochondrial respiration is the oxygen consumption associated with other cellular activities and can be due to enzymatic reactive oxygen species (ROS) production, cell-surface oxygen consumption, and non-mitochondrial NADPH oxidases, including cytochrome P450s [51–55]. Interestingly, lower levels of non-mitochondrial respiration were observed in both the *him-5(e1490)* and *him-8(e1489)* strains (Fig. 3G).

**Fig. 4:**
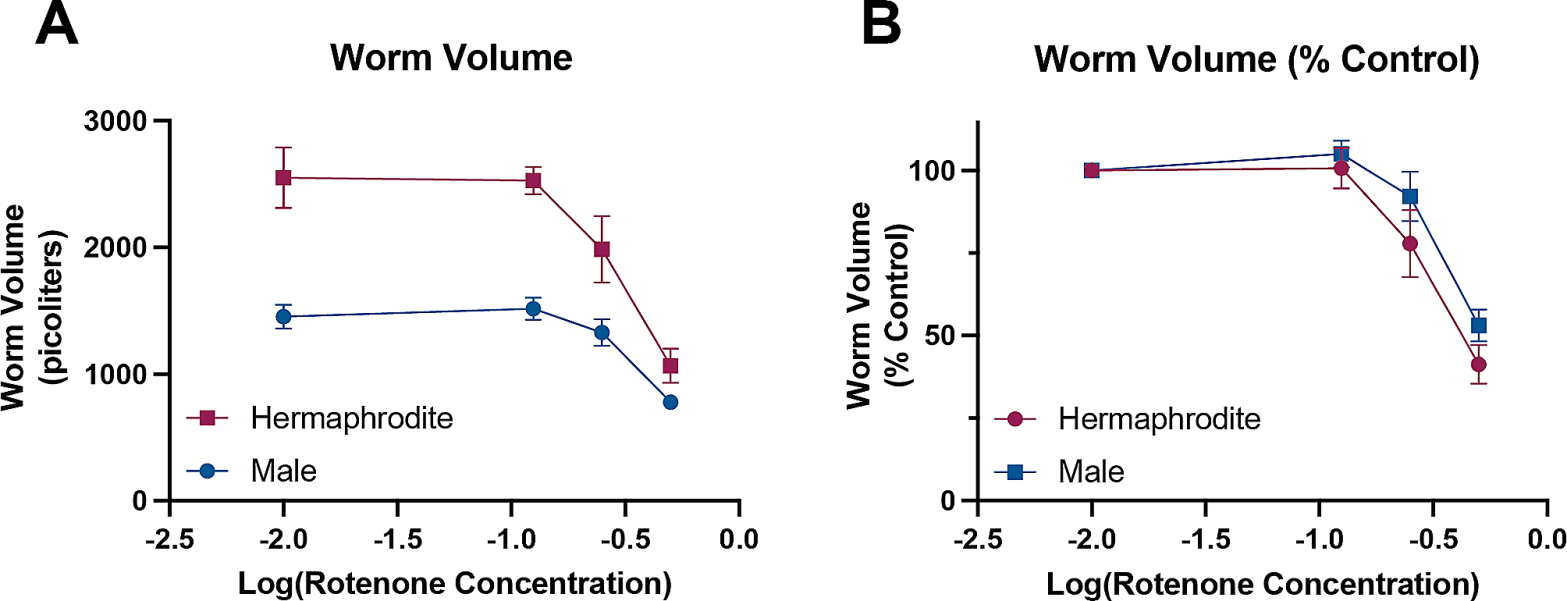
Wild type hermaphrodites and males have similar sensitivity to developmental rotenone exposure. **(A)** Dose response curves for rotenone exposure in males and hermaphrodites expressed in terms of the raw value of the worm volume following a 72-hour exposure to rotenone. Significance was assessed by a two-way ANOVA for sex and dose, sex: ***, *p* < 0.0001, dose: ***, *p* < 0.0001, interaction: *, *p* = 0.0196. **(B)** Dose response curves for rotenone exposure in males and hermaphrodites with worm volume expressed as a percentage of the control. Significance was assessed by a two-way ANOVA for sex and dose, sex: *p* = 0.0685, dose: ***, *p* < 0.0001, interaction: *p* = 0.5749. For both graphs, the x-axis represents the log transform of the rotenone concentration, where the doses were 0, 0.125, 0.25, and 0.5 µM. For the 0 µM dose, a value of 0.01 was used to log transform the data, so -2 represents the vehicle control. Three biological replicates were assessed, each with > 50 worms assessed per replicate

We considered the possibility that sex differences may be latent and only manifest upon challenge. Rotenone is a well-characterized pesticide and a classic mitochondrial toxicant that inhibits Complex I of the electron transport chain. In mammalian models, males have increased sensitivity to rotenone [56]. To assess sex differences in sensitivity to mitochondrial stress, we generated dose-response curves for developmental exposure to rotenone in liquid culture. Growth of *C. elegans* was measured following exposure to various rotenone concentrations by analyzing the total worm volume [57], as mitochondrial inhibition has been shown to impact nematode growth [58]. Rotenone caused growth delays in both male and hermaphrodite wild-type N2 worms (Fig. 4A). There was a significant difference in size between the sexes (*p* < 0.001), as expected, as well as a significant dose effect (*p* < 0.001) with rotenone causing growth delays, as previously reported [59]. Additionally, there was a significant dose by sex interaction term (*p* = 0.0196), with hermaphrodites appearing to be slightly more sensitive to rotenone, as indicated by the larger overall reduction in body size. However, when assessing the influence of rotenone on body size as a function of the sex-specific unexposed control population, i.e., normalizing to the unexposed controls, the sex effect and dose by sex interaction terms become non-significant (Fig. 4B, *p* = 0.0685, *p* = 0.5749 respectively).

**Fig. 5:**
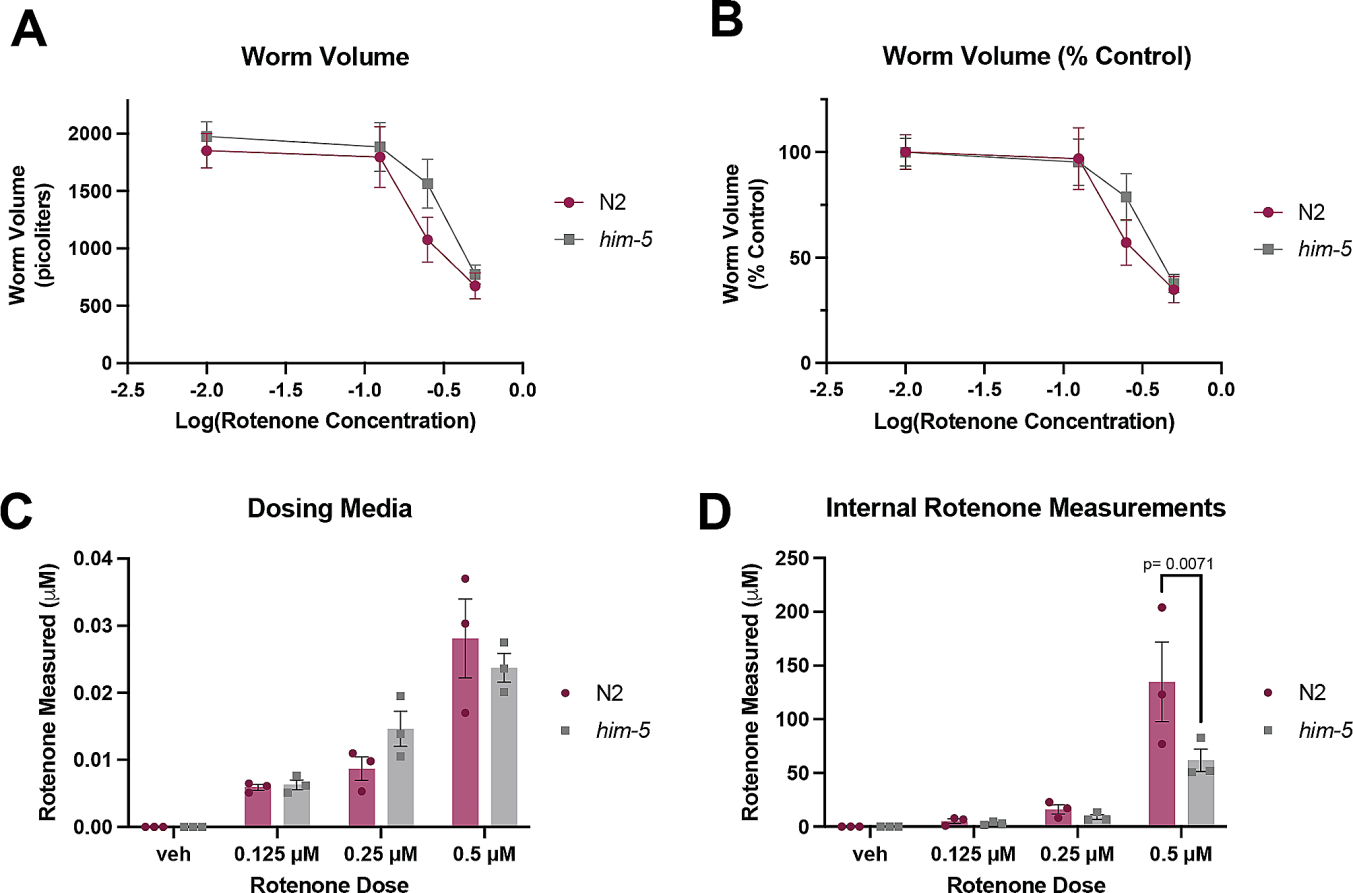
Analysis of rotenone uptake in N2 and *him-5* strains. **(A)** Dose response curves for rotenone exposure in N2 and *him-5* strains expressed in terms of the raw value of the worm volume following a 72-hour exposure to rotenone. Significance was assessed by a two-way ANOVA for strain and dose, strain: *p* = 0.1342, dose: ***, *p* < 0.0001, interaction: *p* = 0.6392. **(B)** Dose response curves for rotenone exposure in N2 and *him-5* strains with worm volume expressed as a percentage of the control. Significance was assessed by a two-way ANOVA for strain and dose, strain: *p* = 0.4153, dose: ***, *p* < 0.0001, interaction: *p* = 0.6118. For graphs in panels A and B, the x-axis represents the log transform of the rotenone concentration, where the doses were 0, 0.125, 0.25, and 0.5 µM. For the 0 µM dose, a value of 0.01 was used to log transform the data, so -2 represents the vehicle control. **(C)** Detected concentrations of rotenone in the dosing media following the 72-hour exposure. On the x-axis is the intended dose and on the y-axis is the detected amount remaining at the time of harvesting worms for analysis. Significance was assessed by a two-way ANOVA assessing strain and dose, strain: *p* = 0.7838, dose: ***, *p* < 0.0001, interaction: *p* = 0.2697. **(D)** Detected internal concentrations of rotenone in the worms following the 72-hour exposure. On the x-axis is the intended dose and on the y-axis is the internal dose calculated using the worm volume determined using Wormsizer. Significance was assessed by a two-way ANOVA assessing strain and dose with a Šidák post hoc test for multiple comparisons, strain: *p* = 0.0513, dose: ***, *p* < 0.0001, interaction: *, *p* = 0.0501. Three biological replicates were assessed

Finally, we assessed if there were sex differences in uptake of the lipophilic compound rotenone between males and hermaphrodites, given known sex differences in *C. elegans* size and metabolism [27–29]. Due to the large numbers of *C. elegans* required for determining rotenone uptake, we were unable to assess uptake in WT hermaphrodite and male populations given the low number of males present and the inability to sort them efficiently. To circumvent this, we repeated our rotenone dose response, comparing N2 WT populations, with no males present in the strain, to *him-5(e1490)*, which is ~ 50% male. We saw no differences in sensitivity to rotenone between these strains (Fig. 5A and B). To ensure that all treatment groups were dosed with similar levels of rotenone, we assessed rotenone concentrations in the dosing medium for each experiment, in which the *C. elegans* were cultured. No statistically significant difference was observed between the measured rotenone in the dosing media of the N2 and *him-5(e1490)* populations at any of the rotenone doses (Fig. 5C). However, we did observe a statistically significant difference in internal rotenone concentrations between N2 and *him-5(e1490)* worms at a rotenone dose of 0.5 µM (*p* < 0.001), with the N2 worms having a higher internal dose of rotenone than the *him-5(e1490)* worms (Fig. 5D). A statistically significant difference was not observed at the 0.125 µM and 0.25 µM doses, although the average rotenone concentration in the N2 worms appeared to be greater than in the *him-5(e1490)* worms at both doses. Interestingly, the rotenone concentrations measured in the worms (Fig. 5D) were greater than the rotenone concentrations measured in the media (Fig. 5C), indicating that rotenone was accumulating within the worms.

## Discussion

Sex differences in mitochondrial function reported in mammals include higher ATP levels [4], lower levels of ROS [16, 60], and greater mtDNA content in females [15]. While these differences have typically been attributed to the effects of sex hormones [61], it is also hypothesized that the pattern of maternal inheritance may also lead to evolutionary selection for improved mitochondrial function in females [15]. Here, we characterized parameters of mitochondrial function in *C. elegans* sexes to assess their utility and limitations as a mitochondrial biology and toxicology model.

We observed sex differences in mtDNA copy number, with hermaphrodites showing higher mtDNA levels than males across all studied strains (Fig. 2). However, this sex difference is expected and likely driven by the presence of developing oocytes in the L4 hermaphrodites [48] rather than sex per se. There were no differences in copy number across the studied strains (Fig. 2). We also observed no differences in whole worm ATP levels or mitochondrial respiration across any of the populations (Figs. 2 and 3).

Mitochondrial DNA copy number remains steady [48] or increases slightly [62] throughout L1-L3 stages, followed by a five-fold increase at the L4 stage in hermaphrodites, and then a six-fold increase in gravid adult hermaphrodites, but only a two-fold increase in males [48]. Though previous work has shown no differences in mtDNA copy number between males and hermaphrodites at the L4 stage [48], we suspect that we are observing these differences due to slight differences in the timing of our experimental design. It is likely that we are capturing mid-L4 staged worms, and previous work may have been assessing early L4 worms, which is supported by the reported fold-change differences from L1 to L4 in the work by Tsang and Lemire [48] compared to our protocol for L4 staged worms [62]. This larval stage is associated with the formation of the germline in hermaphrodites and our mtDNA copy number data suggests we are beginning to observe the formation of mtDNA-rich oocytes. That germline proliferation was occurring is further supported by our nuclear DNA copy number measurements, which (consistent with previous analysis [62, 63]) is ~ 1000 copies/worm higher than can be explained solely by somatic cell number, even including polyploid intestinal and hypodermal cells (959 diploid somatic cells; 34 32-ploid intestinal cells; 98 4-ploid hypodermal cells). Germline proliferation during L4 may also contribute to between-replicate variability observed, as small differences in timing could result in significant differences in DNA copy number. In contrast, the lack of observable differences in whole worm ATP levels or mitochondrial respiration across strains suggests that at this worm stage, oocyte mitochondrial function is very low and that the presence of developing oocytes alone was not enough to stimulate higher mitochondrial bioenergetic function in the hermaphrodites.

The lack of detectable sex differences in markers of basal mitochondrial function, as indicated by whole worm ATP levels and respirometry, indicates similar mitochondrial function in males and hermaphrodites. It is possible that we were unable to distinguish differences in mitochondrial function between *C. elegans* sexes if differences are smaller than what our methods allow us to detect. The inability to culture pure male populations may also limit the level of detection available for the whole worm ATP and respirometry assays. Further, in mammalian systems, many of these sex differences have only been assessed in specific cell or tissue types [15]. Thus, it is possible that sex differences in mitochondrial function exist in *C. elegans*, but, without cell-specific resolution including isolation of somatic cells from the germline, we are unable to detect them. Similarly, we cannot rule out the existence of life stage- or age-specific sex differences, or differential sensitivity to mitochondrial toxicants or stressors other than those we tested.

Nonetheless, while we cannot rule out subtle differences, we did not observe any large sex-specific differences in mitochondrial parameters. The lack of robust sex differences in mitochondrial function in this model system is not consistent with the argument that maternal inheritance of mtDNA contributes significantly to establishing sex differences in mitochondrial function, as *C. elegans* also have maternal inheritance of mtDNA. However, given the current limitations in the ability to culture pure populations of male *C. elegans* and the sensitivity of our methods, we cannot definitively draw this conclusion. Further, the unique nature of sex determination in *C. elegans* and their shift in sex proportions during times of stress prevents a direct extrapolation of our results to mammalian systems. Given that male *C. elegans* are more likely to arise under conditions of stress (temperature, etc.), it remains plausible that in this biological system, males may have elevated fitness in response to specific stressors. Previously, sex differences in the susceptibility to toxicant exposure has been documented. *C. elegans* males were found to be more resistant to methylmercury [64], juglone, and hyperosmolarity [65], but less resistant to arsenic, hydrogen peroxide, paraquat, and ultraviolet light [66]. Thus, our results represent initial investigation into this topic, rather than definitive conclusions, and bring up potential limitations to non-mammalian model organisms when studying sex-variable cellular phenotypes.

To assess whether sex differences arise in the presence of mitochondrial stress, which could reveal otherwise-silent functional deficits, we assessed worm growth following a developmental exposure to a classic mitochondrial toxicant, rotenone. Rotenone inhibits Complex I of the electron transport chain, resulting in decreased ATP production, increased ROS production [67], apoptosis, as well as altered mitochondrial fission and fusion [68]. It has been linked to Parkinson’s disease, a neurodegenerative disease more common in men than women [69]. Sex-specific sensitivities to rotenone have been demonstrated in mammals, with males being more sensitive to rotenone [56]. The results of our study, however, do not demonstrate robust sex differences in rotenone sensitivity in *C. elegans*. Given the need to rapidly screen chemicals for consumer and environmental safety, more researchers are looking towards models like *C. elegans* that allow for rapid and high-throughput in vivo models [39–42]. Our study suggests that *C. elegans* is unlikely to provide insight into mammalian sex-specific toxicologic responses that may occur, particularly when looking for mitotoxic impacts.

Our measurement of several hundred-fold more rotenone in the worms than in the dosing medium suggests significant bioaccumulation, although we did not carry out a time-course to permit measurement of internal worm half-life of rotenone. (Fig. 5C-D, Figure S1). It is not surprising that the rotenone concentration in the dosing medium is low, as the reported half-life of rotenone in 24 °C water is 13.9 h [70]. Rotenone is a highly lipophilic compound and may be stabilized in lipophilic compartments in the organism, which could protect rotenone from degradation and lead to the bioaccumulation of the compound. Interestingly, we did observe higher internal concentrations of rotenone in N2 worms than the *him-5(e1490)* male enriched population of worms when exposed to 0.5 µM rotenone (Fig. 5D). Given that rotenone is a lipophilic compound we suspect that the elevated internal dose in N2 worms may be because rotenone accumulated in the oocytes given their high lipid content [71]. It is also possible that the oocyte lacks high xenobiotic metabolism capacity, although this has not to our knowledge been formally tested. Future work should assess accumulation of rotenone and other lipophilic toxicants in the eggs specifically as this may have important implications for *C. elegans* use as a toxicological model.

Of additional note, we observed reduced non-mitochondrial respiration in the *him-5(e1490)* and *him-8(e1489)* strains (Fig. 3G). This reduction was likely driven by a strain difference rather than sex difference because reduced non-mitochondrial respiration was not observed in the N2 male enriched population or *fog-2(q71)* strain. Non-mitochondrial respiration in cells can be driven by biological processes such as enzymatic ROS production, cell-surface oxygen consumption, and non-mitochondrial NADPH oxidases, including cytochrome P450s [51–55]. *him-5(e1490)* and *him-8(e1489)* strains are often used by *C. elegans* researchers studying males. Our data suggests that these mutations which yield male enriched populations may have secondary effects on one or more of these biological processes, which should be considered when using these strains.

## Conclusion

We did not observe robust sex differences in mitochondrial function or in response to a mitochondrial toxicant in *C. elegans.* While further work is needed to assess an evolutionary justification for sex differences in mitochondrial function arising from the pattern of maternal inheritance, our data does not support a large effect of mito-nuclear crosstalk. Finally, this study also highlights a limitation of *C. elegans* as a toxicological and mitochondrial health model, given that the worms’ response to a mitochondrial toxicant did not mirror the sex-specific response observed in mammalian systems.

## Methods

### Worm maintenance

All strains of *C. elegans* used in this study were maintained on K-agar plates and fed the OP50 strain of *E. coli* at 20 °C. *C. elegans* strains in this study were wild type (N2), *fog-2(q71)*, *him-5(e1490)*, *and him-8(e1489)*, all of which were acquired from the *Caenorhabditis* Genetics Center, University of Minnesota. To generate male enriched N2 populations (“N2ME”), 60 mm mating plates were set up at a 15:5 hermaphrodite: male ratio and egg prepped five days later. The *him-8* and *him-5* mutations cause X chromosome nondisjunction, resulting in higher incidence rates of males. The *fog-2* mutation results in the inability for hermaphrodites to produce sperm, making them dependent on sexual reproduction and thus maintaining higher frequencies of males. The frequency of males in each strain used can be found in Fig. 1.

### Male frequency determination

To determine the proportion of males in *C. elegans* populations, staged L4 worms (*n* > 75 per replicate) were plated on food-free K-agar plates and imaged using a Keyence BZ-X710 using the stitching feature. Stitched images of plates were then used to manually count the number of males and hermaphrodites in the population. The frequency of males in each population was performed alongside each assay that relied on entire populations, i.e., ATP quantification and Seahorse Respiration assays and thus represent the proportion of males present at the time of each experiment.

### Egg prep/synchronization of *C. elegans*

Synchronized populations of L1 worms for each experiment were obtained through a hypochlorite/NaOH treatment of gravid adults to harvest eggs [72]. Embryos were allowed to hatch for up to 16 h in K + medium, K-medium supplemented with cholesterol [73]. Synchronized larval stage 1 (L1) larvae were then plated on k-agar plates supplemented with OP50 *E. coli* at 20 °C for 48 h to generate L4 worms for respirometry analysis, mtDNA copy number, and ATP levels.

### ATP quantification

ATP levels were measured using the CellTiter-Glo Luminescent Cell Viability Assay (Promega G7572), and normalized to protein content determined by the Pierce bicinchoninic acid assay (Thermo Scientific, Rockford, IL) [74]. Briefly, 200 synchronized L4 worms were collected, flash frozen and stored at -80 °C. At the time of analysis, worm samples were boiled at 95 °C for 15 min and centrifuged to remove cellular debris. Aliquots of the worm extractions were used for ATP quantification and total protein determination. ATP content was normalized to total protein. In total, four biological replicates were performed.

### mtDNA copy number analysis

Six synchronized L4 worms of each sex and each strain were picked into 90 µL of worm lysis buffer (25 mM Tricine, pH 8; 80 mM potassium acetate; 11% w/v glycerol; 2.25% v/v DMSO, 1 mg/mL proteinase K in nuclease-free water), flash frozen, and stored at -80 °C. At the time of analysis, samples were lysed at 65 °C for 1 h, and the lysate was used as a template in real-time PCR experiments [75]. Power Sybr Green PCR Master Mix (ThermoFisher Scientific, Waltham, MA) was used with 2 µL of lysate for the template. CT values were converted to copy number using a standard curve with the pCR 2.1 plasmid containing the species-specific mitochondrial *nduo-1* gene fragment for mtDNA or the *cox-4* nuclear gene fragment. Copy number was calculated per worm. Primers and PCR conditions can be found in Supplemental Material Table 1. PCR was run in technical triplicate and in total, three biological replicates were performed.

### Seahorse respiration assays

Synchronized L4 worms of each strain were seeded into a Seahorse XFe24 Extracellular Flux Analyzer microplate at a density of 75 worms/well, as described previously [76] and subjected to a modified version of the “mitochondrial stress test”. Briefly, basal oxygen consumption rate (OCR) measurements are taken before injection of either 25 µM (final) carbonyl cyanide 4-(trifluoromethoxy)phenylhydrazone (FCCP, a mitochondrial uncoupler) to measure maximal respiration or 20 µM N, N-dicyclohexylcarbodiimide (DCCD, an ATP synthase inhibitor), to determine ATP-linked respiration). After injection of either FCCP or DCCD, 14 measurements were performed before a final injection of 10 mM sodium azide (a complex IV inhibitor) to completely inhibit mitochondrial respiration and determine the non-mitochondrial OCR. Final parameters calculated include Basal Mitochondrial OCR (Basal OCR– non-mitochondrial OCR), Spare Capacity (Maximal OCR– Basal OCR), ATP-linked Respiration (Basal OCR– DCCD-inhibited OCR), and Proton Leak (DCCD-inhibited OCR– non-mitochondrial OCR). Seahorse experiments included 5 wells per treatment group (technical replicates) and at least 5 biological replicates. Seahorse experiments were normalized to worm volume, determined by imaging a subset (*n* > 30 worms) per group using the ImageJ plugin WormSizer [57].

### Developmental rotenone exposure

Synchronized L1 larvae were generated as previously described for other experiments. Given the short half-life of rotenone and uncertainty about uptake of compounds into worms from agar plates, exposure to rotenone was performed in liquid culture. Following hatching of embryos, L1 larvae were counted and 500 worms per well were transferred into liquid culture in 6-well plates containing K+, metabolically inactive food (UVRA *E. coli* [77]) and the chemical exposure of either a DMSO control, 0.125 µM, 0.25 µM, or 0.5 µM rotenone. Given that rotenone has a short half-life and degrades rapidly, each group was re-dosed with rotenone every 24 h. After a 72-hour exposure, worms (*n* > 50 per group) were placed on food-free K-agar plates and imaged using a Keyence BZ-X710. The worm volume was then calculated using the ImageJ plugin WormSizer [57]. Worm volume was normalized to the control group and represented as a percentage of the control volume size, wherein 100% was defined as the volume of the control group and 0% was defined as 48 picoliters, the mean volume of an L1 [57]. In total, three biological replicates were performed.

### Rotenone uptake and internal dose quantification

Rotenone uptake was assessed in wild type N2 and the male enriched strain, *him-5*. For uptake measurements, rotenone exposures were carried out for 72 h as in other measurements. For each individual replicate, 1000 worms were exposed to either a DMSO control, 0.125 µM, 0.25 µM, or 0.5 µM rotenone for 72 h in liquid culture as previously described. Following exposure, samples were transferred to 2 mL centrifuge tubes and the worms were pelleted at 600 x g. The supernatant was removed and saved for analysis, known as ‘dosing media’ as this was the liquid medium used to expose worms to rotenone. The worms were washed a total of 3 times as quickly as possible by adding 2 mL K-medium and centrifuging at 600 x g. Following the last spin, the liquid was removed, and the worm pellet was flash-frozen in liquid nitrogen. A total of three biological replicates were performed.

Frozen supernatant samples were thawed at ambient lab temperature (~ 20 °C). Once thawed, 2 µL of the supernatant was spiked with 0.05 ng of d5-atrazine as an internal standard and 1.95 mL of acetonitrile. The samples were vortexed for 30 s, transferred to muffled, amber-glass autosampler vial, capped and stored at -20 °C until analysis.

Frozen worm pellets were thawed at ambient room temperature. Once thawed 0.05 ng of d5-atrazine as an internal standard was spiked into the vial. The worms were extracted with 0.95 mL of acetonitrile via bath sonication for 15 min at room temperature, the sample was then centrifuged (10 min, 15,000 RPM, 4 °C). The acetonitrile supernatant was pipetted into an autosampler vial, and an additional round of extraction was completed. Both supernatants were combined and stored at -20 °C until analysis.

Analysis by LC-MS/MS was done within 20 h of sample processing. Separation was done using a Vanquish ultra-high pressure liquid chromatograph (UHPLS; ThermoFisher, San Jose, CA, USA) coupled to a TSQ Altis triple quadrupole mass spectrometer (ThermoFisher, San Jose, CA, USA) detector. Chromatography was achieved with a 5 µL injection onto a 100 × 2.1 mm, 1.9 μm particle size, Hypersil Gold aQ C18 (ThermoFisher, San Jose, CA, USA), analytical column with a flow rate of 0.3 mL/minute and temperature of 35 °C. Mobile phases were water and acetonitrile both spiked with 0.1% formic acid and the gradient was held at 90% aqueous for 2 min, then ramped to 100% organic over 9 min and held for 30 s, before ramping back to 90% aqueous for a total time of 14 min. Detection was done using electrospray ionization in positive mode with a capillary potential of 3500 V. The vaporizer temperature and ion transfer tube temperature were held at 300 °C and 325 °C, respectively. Selected reaction monitoring (SRM) was used to monitor the following transitions: rotenone: 395.1→192.0 and 395.1→213.0 and d5-atrazine 221.1→101.1 and 221.1→179.1. D5-Atrazine was used as an internal standard for quantification which was done using a seven-point calibration curve.

### Statistics

Statistical analysis was performed in GraphPad Prism 9.0. Differences in mean steady state ATP levels and Seahorse XFe24 Extracellular Flux Analyzer parameters were analyzed via a one-way ANOVA when normally distributed, as determined by a Shapiro-Wilk normality test, with Tukey’s HSD post-hoc tests used for multiple comparisons. When data was not normally distributed, parameters were analyzed using a Kruskal-Wallis test with a Dunn’s post-hoc test for multiple comparisons of each group to one another. Differences in mtDNA and nuclear DNA copy number were assessed via two-way ANOVA to assess effects of sex and strain. Statistical significance for both dose response and rotenone uptake studies was assessed by a two-way ANOVA for sex and dose with a Šidák post hoc test for multiple comparisons. Significance was determined by *p* < 0.05. For all graphs, error bars represent the standard error of the mean (SEM).

### Electronic supplementary material

Below is the link to the electronic supplementary material.

Supplementary Material 1

## Data Availability

The datasets used and analyzed in this study are available from the corresponding author on reasonable request.

## Abbreviations

mtDNA: Mitochondrial DNA
OCR: Oxygen Consumption Rate
N2ME: N2 Male Enriched Population
