# Supplementary material for: Lack of detectable sex differences in the mitochondrial function of Caenorhabditis elegans
Source: BMC Ecol Evol. 2024 Apr 26;24:55. doi: 10.1186/s12862-024-02238-x (PMC11046947; doi:10.1186/s12862-024-02238-x)
Supplement: Supplementary file 1 — Supplementary Material 1 [file 12862_2024_2238_MOESM1_ESM.docx]

**SUPPLEMENTARY INFORMATION**

**Lack of detectable sex differences in the mitochondrial function of *Caenorhabditis elegans***

Dillon E. King^1,2^ *, A. Clare Sparling^1^ *, Abigail Joyce^3^, Beverly DeSouza^4^, P. Lee Ferguson^3^, Susan K. Murphy^1,2^, and Joel N. Meyer^1‡^

*Contributed equally

*^1^Nicholas School of Environment, Duke University*

*^2^Department of Obstetrics and Gynecology, Duke University Medical Center*

*^3^Pratt School of Engineering, Duke University*

*^4^Department of Pharmacology and Cancer Biology, Duke University*

‡ Correspondence:

308 Research Drive, A304

Durham, NC 27708

(919) 613-8109

[joel.meyer@duke.edu](mailto:joel.meyer@duke.edu)

**Table S1. Primers and PCR Conditions for Copy Number Analysis**

| **Genome** | **Forward Primer** | **Reverse Primer** | **T_Anneal_ (°C)** | **Gene** | **Amplicon Size (bp)** |
| --- | --- | --- | --- | --- | --- |
| Mitochondrial | 5’-AGC GTC ATT TAT TGG GAA GAA GAC-3’ | 5’-AAG CTT GTG CTA ATC CCA TAA ATG T-3’ | 60 | *nduo-1* | 75 |
| Nuclear | 5’-GCC GAC TGG AAG AAC TTG TC-3’ | 5’-GCG GAG ATC ACC TTC CAG TA-3’ | 60 | *cox-4* | 164 |

**
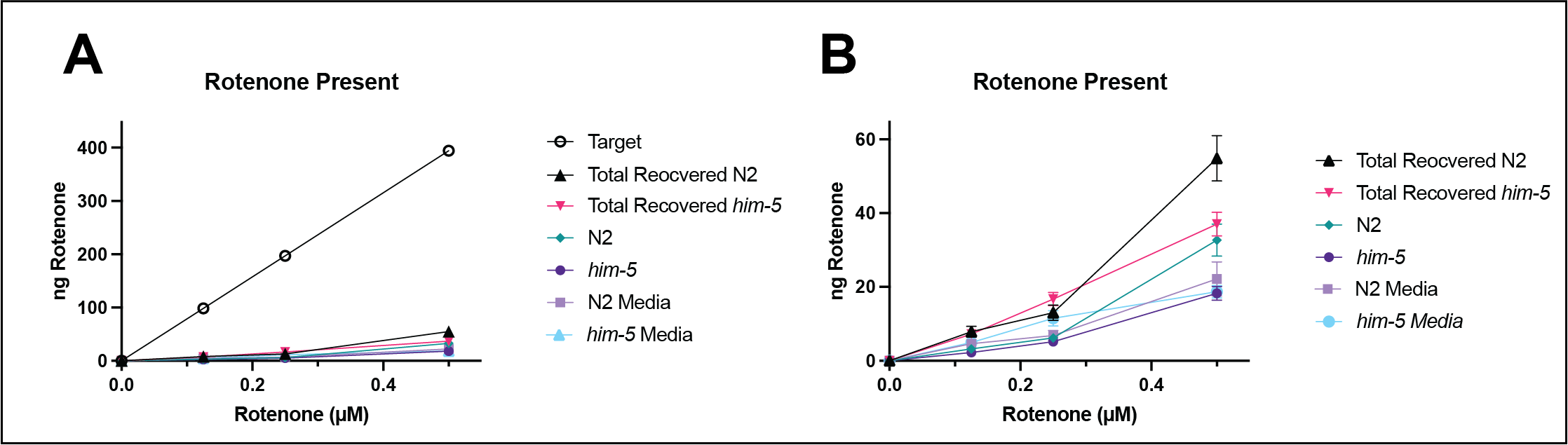
**

**Figure S1:** **Mass balance analysis of rotenone uptake in N2 and *him-5* strains.** A) Detected levels of rotenone present (ng) of each sample matrix. The ‘target’ level represents the amount of rotenone expected to be present in 2 mL of dosing media at each dose. The ‘total recovered’ value for each strain is the sum of the total amount of rotenone present in the worm sample and the total amount present in the 2 mL of dosing media. B) Panel B is the same figure as panel A, without the target levels of rotenone for visualization purposes. In both panels, the y-axis represents the ng of rotenone present, and the x-axis represents the concentration of rotenone the samples were dosed with.
